# Directed functional connectivity of the default-mode-network of young and older healthy subjects

**DOI:** 10.1038/s41598-024-54802-6

**Published:** 2024-02-21

**Authors:** Gadi Goelman, Rotem Dan, Ondrej Bezdicek, Robert Jech, Dana Ekstein

**Affiliations:** 1 Department of Neurology, Ginges Center of Neurogenetics, Hadassah Hebrew University Medical Center, 91120 Jerusalem, Israel; 2 Faculty of Medicine, The Hebrew University of Jerusalem, Jerusalem, Israel; 3 Edmond and Lily Safra Center for Brain Sciences, The Hebrew University of Jerusalem, Jerusalem, Israel; 4 Department of Neurology and Center of Clinical Neuroscience, Charles University, Prague, Czech Republic

**Keywords:** Aging, Directed functional connectivity MRI, Default mode network, Multivariate analysis, Neuropsychological tests, Cognitive ageing, Neural ageing, Neural circuits, Sensorimotor processing, Neurology

## Abstract

Alterations in the default mode network (DMN) are associated with aging. We assessed age-dependent changes of DMN interactions and correlations with a battery of neuropsychological tests, to understand the differences of DMN directed connectivity between young and older subjects. Using a novel multivariate analysis method on resting-state functional MRI data from fifty young and thirty-one healthy older subjects, we calculated intra- and inter-DMN 4-nodes directed pathways. For the old subject group, we calculated the partial correlations of inter-DMN pathways with: psychomotor speed and working memory, executive function, language, long-term memory and visuospatial function. Pathways connecting the DMN with visual and limbic regions in older subjects engaged at BOLD low frequency and involved the dorsal posterior cingulate cortex (PCC), whereas in young subjects, they were at high frequency and involved the ventral PCC. Pathways combining the sensorimotor (SM) cortex and the DMN, were SM efferent in the young subjects and SM afferent in the older subjects. Most DMN efferent pathways correlated with reduced speed and working memory. We suggest that the reduced sensorimotor efferent and the increased need to control such activities, cause a higher dependency on external versus internal cues thus suggesting how physical activity might slow aging.

## Introduction

Cognitive decline, reduced processing speed, difficulties with attention and with working and episodic memory are common findings associated with healthy aging^1–5^. Studies using multiple connectivity measures of healthy young and older subjects have shown reduced default mode network (DMN) connectivity and an altered functional organization of many primary sensory and cognitive networks in the later^1^. The DMN is best known for being active during wakeful rest, such as daydreaming or mind-wandering. It is also active when an individual is thinking about others, thinking about themselves, remembering the past, and planning the future^6^. Changes in the DMN have been shown to be associated with neurological and psychiatric diseases, e.g. it is vulnerable to the early stages of Alzheimer disease (AD) pathology^7,8^.

Within the DMN, the posterior cingulate cortex (PCC) has been shown to be functionally segregated, with its ventral part primarily connected to other DMN regions and supporting internally directed thought^9^ while its dorsal part is primarily connected to multiple networks including the sensorimotor network and supports externally directed attention^10^. Subjects with AD and with amnestic mild cognitive impairment (aMCI) have been shown to have a modulated PCC connectivity. While the ventral PCC network was atrophied in aMCI and AD patients, the dorsal network was only atrophied in AD patients^11^. It is, therefore, of great clinical relevance to understand the functionality of the dorsal and ventral PCC in elderly subjects.

Connectivity of the DMN has been addressed in many resting-state functional magnetic resonance imaging (rs-fMRI) studies in young and old subjects however, with inconsistent findings. Some have shown reduced anterior–posterior intra DMN connectivity^12^, while others have revealed a complex increase and decrease in the connectivity of specific connections^13–17^. We suggest that this inconsistency may be attributed to the use of bivariate and undirected connectivity measurements. Consider, for example, the connectivity between the PCC and the supplementary motor area. If these regions are involved in two separated information flows such as: mPFC → PCC → SMA and SMA → PCC → Angular, the bivariate undirected connectivity between the PCC and the SMA gives an average coupling which is an inaccurate estimation of the functional coupling between these regions.

To derive a more precise connectivity estimation, we recently introduced a multivariate analysis that can be used with rs-fMRI data. This innovative approach facilitates the identification of interactions and directionality among four distinct anatomical regions using phase coherence. Specifically, it allows for the delineation of functional directed couplings and pathways, enabling the identification of directed interactions among these regions to define anterograde information flow pathways^18–23^. Computer simulations of the Kuramoto model have tested the accuracy of this analysis^18^, and its applications to the human brain were demonstrated in the above sited studies.

We propose that DMN connectivity varies with age and correlates with the declining cognitive and adaptive abilities observed in older individuals. Specifically, we aim to investigate how age impacts the functionality of the dorsal and ventral PCC. To test these hypotheses, we applied our multivariate functional connectivity analysis to rs-fMRI data.

Our approach involved identifying functional directed pathways within the DMN (intra-DMN) and between the DMN and other brain regions (inter-DMN), comparing 50 healthy young participants with 31 healthy older individuals. Furthermore, we sought to elucidate the behavioral implications of these pathways by examining their associations with a battery of neuropsychological tests.

## Theory

A detailed description of the analysis was presented in our previous publications^18–23^, therefore, only the main points are summarized below. For a group of four weakly coupled rs-fMRI BOLD temporal signals, with each signal corresponding to a different anatomical location, the analysis assumed that the phases contained information of the temporal order of their mutual coupling. This order is expressed in terms of specific relations between the four phases^18^, and enables the definition of four-node directed pathways corresponding to information transfer among them. For resting-state data, we averaged over time, in the time–frequency wavelet space, to obtain frequency-dependent phase differences. We restricted the analysis to continuous, effectively unidirectional pathways to define pathways among the four BOLD signals, i.e., pathways that started in one region and subsequently went through all the other regions. In this case, there were 24 possible pathways (listed in Supplementary Table 1). In this table, three of the four regions were symbolized by R1 to R3, while the fourth region was symbolized as “X”. By choosing pathways that were invariant to the choice of reference-phase, we guaranteed that all phase differences were below 2π^21,22^, thus obtaining unbiased pathways.

For each participant (*sub*), each pathway (from Supplementary Table 1) (*k*) and each frequency ($\omega ),$ a binary pathway value (*PW*) was defined as “1” for the cases where phase differences were in line with the pathway and “0” for when they were not (see^21^ and Supplementary Fig. 1 their):

1 $$PW^{k} \left( {\omega ,sub} \right) = \left\{ {\begin{array}{*{20}c} 1 \\ 0 \\ \end{array} } \right\}\begin{array}{*{20}c} {phases\;in\;line\;with\;the\; k\; pathway\;fo\;r all \;4 \;reference \;phases} \\ {phases{ }\;not{ }\;in\;{ }line{ }\;with{ }\;the{ }\;pathway} \\ \end{array}$$

with ‘k’ = 1, 2… 24 corresponding to a pathway's number in Supplementary Table 1, “$\upomega$ ” the frequency scale, and “*sub*” a subject. Note that , $P{W}^{k}\left(\omega ,sub\right)=1$ only for one of the kth, while for all the others it equaled zero.

A group pathway index (PWI) was defined as:

2 $$PW{I}^{k}\left(\omega \right)=\frac{1}{N}\sum_{i=sub}^{N}P{W}^{k}\left(\omega ,sub\right),$$

similar to the definition of the phase lag index (PLI)^24,25^ but describing the coherence among four regions, while PLI describes the coherence between two regions. We further note that averaging the wavelet coherences among participants solved the intrinsic time–frequency uncertainty^26,27^.

Comparisons between groups were performed as follows:

3 $$\Delta PW{I}^{k}\left(\omega \right)=PW{I}_{Young}^{k}\left(\omega \right)-PW{I}_{Old}^{k}\left(\omega \right),$$

whose significance was obtained by non-parametric permutation tests.

## Results

Owing to the large number of possible pathways where each fMRI voxel is a region; we first used the Atlas of Intrinsic Connectivity of Homotopic Areas (AICHA)^28^ to narrow down the number of regions. Additionally, we devised a strategy to further reduce the number of the pathways, enabling a meaningful comparison between pathways and groups. The detailed description of this strategy can be found in the method section's experimental flowchart and is comprised of four distinct stages. The first stage involved identifying significant between-group pathways using three predefined DMN regions (R1 to R3, as outlined in Supplementary Table 1). In the second stage, we pinpointed regions outside the DMN that played a substantial role in the aforementioned between-group pathways (referred to as the 'X' regions in Supplementary Table 1). This step led to the identification of 18 regions, distributed as follows: six within the visual system, six within the limbic system, and six within the sensorimotor system. In the third stage, we focused on identifying between-group inter-DMN pathways involving these three systems. To gauge the weight of these between-group inter-DMN pathways, we calculated the occurrences of three-node functional pathways. Specifically, since four-node pathways are binary in nature, we calculated the occurrences of three-node pathways by summing the number of pathways, irrespective of whether they included AICHA mPFC or vPCC sub-regions or any of the six visual/limbic or sensorimotor regions. It is these three-node pathways that are presented in our findings and form the basis for our conclusions.

The three preselected regions of the intra-DMN pathways were: the medial prefrontal cortex (mPFC), the posterior cingulate cortex (PCC) and the angular gyrus. The three preselected regions of the inter-DMN were: the mPFC, the PCC and the any of the visual, the limbic or the sensorimotor systems. Furthermore, frequencies were averaged to: low (0.022 Hz, termed scale 1), intermediate (0.04 Hz, termed scale 2), and high (0.073 Hz, termed scale 3).

A three-node directed pathway has six node permutations. Since we differentiated between the ventral and the dorsal PCC (but averaged the mPFC, the angular gyrus and the visual, limbic and sensorimotor systems, see Methods), we had 12 node permutations in each pathway. In the figures and tables below, we present between-groups occurrences of these 12 node permutations for intra-DMN pathways and for inter-DMN pathways. The occurrences of one-group pathways are given in the supplementary. Note however, that the same three-node pathways could result from different four-node pathways. This is since the fourth node could be at different locations, and since each of the three nodes resembles one of several possible regions (see Methods). For all pathways, significant results were only obtained at the two lower frequency scales.

### Intra-DMN pathways

**Figure 1:**
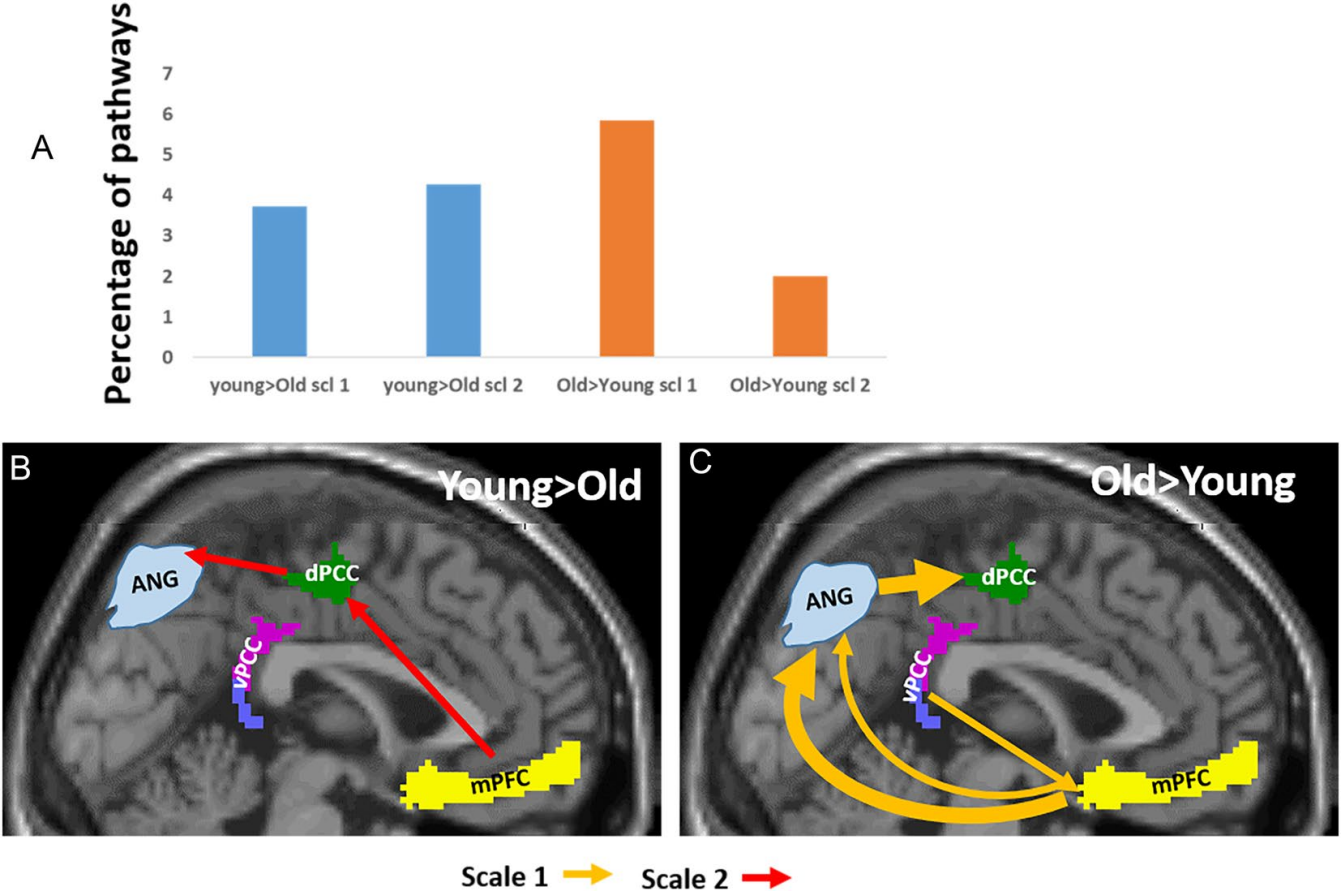
Three-node between-groups functional intra-DMN pathways. (**A**) Percentiles of pathways (out of the maximum possible numbers) that were stronger in the young participants’ group (in blue) and in the older participants’ group (in orange) in scale 1 and scale 2. (**B–C)** Illustrations of pathways that were stronger in the young participants (**B**) and were stronger in the older participants’ group (**C**), with pathways in light orange for scale 1 and in red for scale 2. ‘mPF’C is the medial prefrontal cortex, ‘vPCC’ and ‘dPCC’ are the ventral and dorsal posterior cingulate cortex and ‘ANG’ is the angular gyrus. Only pathways whose occurrences were > 1% are shown with arrow width corresponding to pathway’s occurrences.

Table 1A gives the between-group occurrences of the 12 three-node directed pathways within the DMN in percentage of the maximum number. The numbers of pathways with positive ('Young > Older'; implying stronger connectivity in young than in older subjects) and negative ('Older > Young') $\Delta PW{I}^{k}\left(\omega \right),$ were comparable. However, while more Young > Older pathways were at higher frequency (scale 2), most of the Older > Young pathways were at the lowest frequency (scale 1; 76%) as illustrated in Fig. 1. Furthermore, most of the Older > Young pathways were connected with the dPCC (68%).

**Table 1:** Percentages of significant 3-node between-group pathways (out of the maximum numbers). A. The 12 intra-DMN pathways. B. The 12 inter visual-DMN pathways. C. The 12 inter limbic-DMN pathways. D. The 12 inter sensorimotor-DMN pathways. bold-italic-bolditalic-and underline indicating decreasing occurrences.

	Young > Old		Old > Young	
	Scale 1	Scale 2	Scale 1	Scale 2
(A)				
mPFC > dPCC > Ang		1.06	0.53	
mPFC > vPCC > Ang		0.66		0.93
mPFC > Ang > dPCC			***3.19***	0.53
mPFC > Ang > vPCC	0.93			
dPCC > mPFC > Ang	0.80	0.53		
vPCC > mPFC > Ang			1.06	
dPCC > Ang > mPFC				
vPCC > Ang > mPFC				
Ang > mPFC > dPCC				
Ang > mPFC > vPCC		0.93		
Ang > dPCC > mPFC	0.53			
Ang > vPCC > mPFC	0.80			
Sum	3.06	3.18	4.78	1.46
(B)				
mPFC > dPCC > Visual				
mPFC > vPCC > Visual	1.47	***3.22***	1.14	0.80
mPFC > Visual > dPCC		0.67	2.01	0.54
mPFC > Visual > vPCC	1.54	***3.69***		
dPCC > mPFC > Visual	0.80			
vPCC > mPFC > Visual			0.74	
dPCC > Visual > mPFC	0.54			
vPCC > Visual > mPFC				
Visual > mPFC > dPCC			1.61	
Visual > mPFC > vPCC		1.68		
Visual > dPCC > mPFC				
Visual > vPCC > mPFC	1.01			
Sum	5.36	9.26	5.5	1.34
(C)				
mPFC > dPCC > Limbic				
mPFC > vPCC > Limbic		1.01	0.54	
mPFC > Limbic > dPCC			1.34	
mPFC > Limbic > vPCC		1.34		
dPCC > mPFC > Limbic	0.54	0.67		
vPCC > mPFC > Limbic			0.54	
dPCC > Limbic > mPFC				
vPCC > Limbicl > mPFC				
Limbic > mPFC > dPCC				
Limbic > mPFC > vPCC		1.47		
Limbic > dPCC > mPFC				
Limbic > vPCC > mPFC				
Sum	0.54	4.49	2.42	0
(D)				
mPFC > dPCC > motor			**8.04**	2.68
mPFC > vPCC > motor			*4.09*	1.81
mPFC > motor > dPCC	0.67		0.54	1.47
mPFC > motor > vPCC		0.87		
dPCC > mPFC > motor				
vPCC > mPFC > motor			***3.62***	
dPCC > motor > mPFC	1.07	0.67		1.07
vPCC > motor > mPFC				
motor > mPFC > dPCC				
motor > mPFC > vPCC		1.34		
motor > dPCC > mPFC	2.55			
motor > vPCC > mPFC	0.94			
Sum	5.23	2.88	16.29	7.03

### Inter-DMN-visual pathways

**Figure 2:**
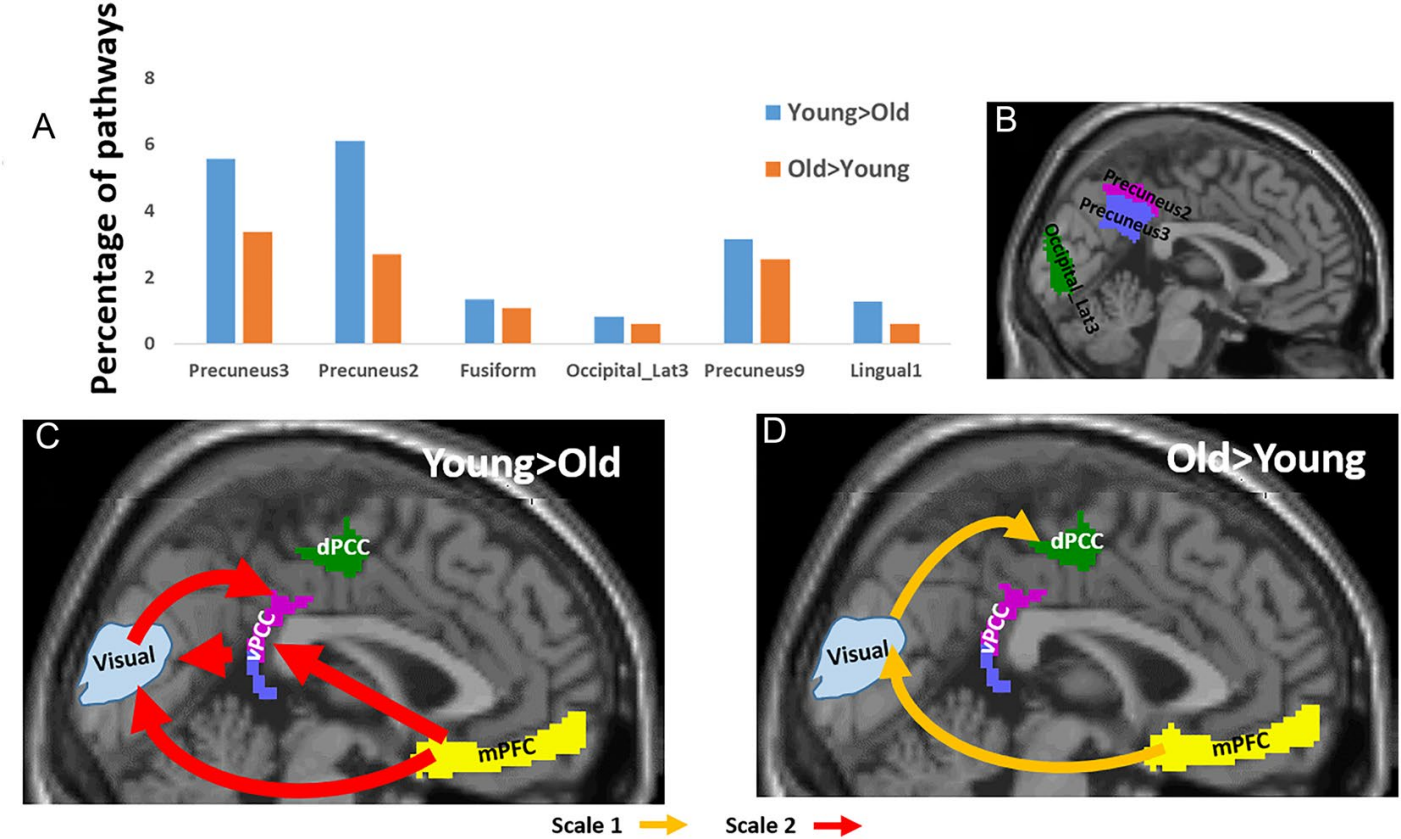
Three-node between-groups functional inter-DMN-visual pathways. (**A**) Percentiles of the pathways (BOLD frequency scales 1 and 2 together) that include a visual region. In blue, pathways that were stronger in the young participants’ group, and in orange, pathways that were stronger in the older participants’ group. (**B**) A 2D sagittal projection of the three visual regions whose pathways differed most between the age groups. (**C–D**) Illustrations of the most dominant pathways that were stronger in the young participants (**C**) and were stronger in the older participants group (**D**), with pathways in light orange for BOLD frequency scale 1, and in red for scale 2. Only pathways whose occurrences were > 2% are shown with arrow width corresponding to pathway’s occurrences.

Table 1B and Fig. 2 gives the between-group occurrences of the 12 three-node directed pathways for pathways connecting the DMN with vision regions. These pathways involved mainly the ventral and dorsal precuneus and to a slighter extent the lingual, the lateral occipital gyri and the fusiform gyrus (Fig. 2A). Specifically, the following six AICHA regions were found in the inter-DMN-visual pathways between group comparisons: Precuneus 3, Precuneus 2, Precuneus 9, Fusiform, Occipital_lat 3 and Lingual 1. As shown in Table 1B, the number of pathways with positive (‘Young > Older’) $\Delta PW{I}^{k}\left(\omega \right)$ values was higher (68%) compared to the number of pathways with negative values ('Older > Young'). Furthermore, while 63% of the Young > Older pathways were at frequency scale 2, 80% of the Older > Young pathways were at scale 1 suggesting age dependence of the BOLD signal frequency. In addition, 79% of the Young > Older pathways involved the vPCC, while 61% of the Older > Young pathways the dPCC.

### Inter-DMN-limbic pathways

**Figure 3:**
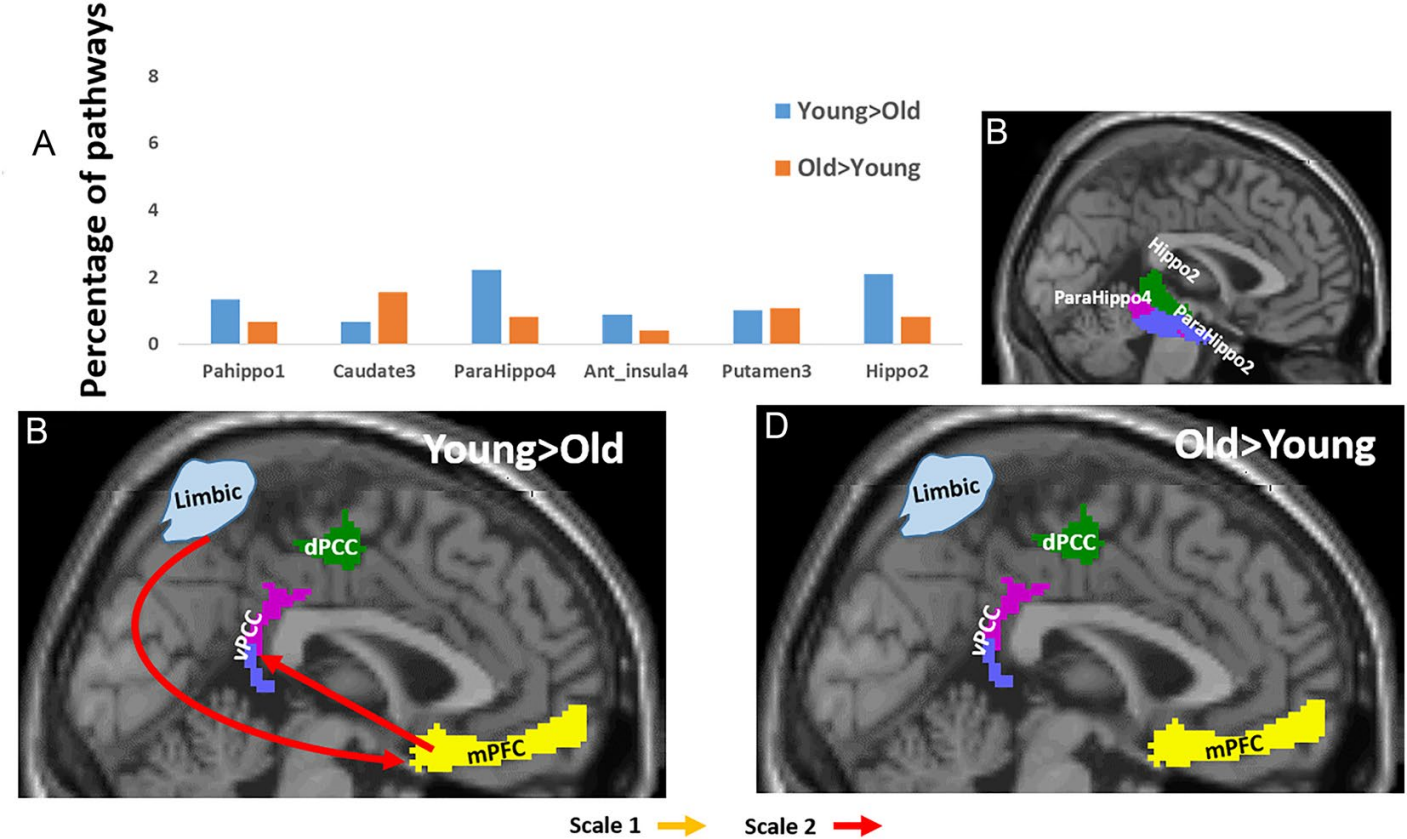
Three-node between groups functional inter-DMN-limbic pathways. (**A**) Percentiles of pathways (BOLD frequency scales 1 and 2 together) that included a limbic region. In blue, pathways that were stronger in the young participants’ group, and in orange, pathways that were stronger in the older participants’ group. (**B**) A 2D sagittal projection of the three limbic regions whose pathways differed most between the age groups. (**C–D**) Illustrations of the most dominant pathways that were stronger in the young participants (**C**) and were stronger in the older participants group (**D**), with pathways in light orange for scale 1, and in red for scale 2. Since the occurrences of all pathways were low, only one whose occurrence is ~ 1% is shown.

Table 1C and Fig. 3 gives the between-group occurrences of the 12 three-node directed pathways for pathways connecting the DMN with limbic regions. Inter-DMN-limbic pathways that were different between the age groups involved the medial parahippocampus, the adjacent hippocampus and, to a slighter extent, the putamen, the anterior insula and the caudate (Fig. 3A). Specifically, the following six AICHA regions were found: Parahippocampus 1, Caudate 3, Parahippocampus 4, Anterior Insula 4, Putamen and Hippocampus 2. The number of pathways with positive $\Delta PW{I}^{k}\left(\omega \right)$ values was higher (67%) compared to the number of pathways with negative values ('Older > Young'). Furthermore, while 89% of the Young > Older pathways were at scale 2, only pathways at scale 1 were significant for the Older > Young group. In addition, 76% of the Young > Older pathways involved the vPCC, whereas the Older > Young pathways were similarly with both PCC sub-regions.

### Inter-DMN-sensorimotor pathways

**Figure 4:**
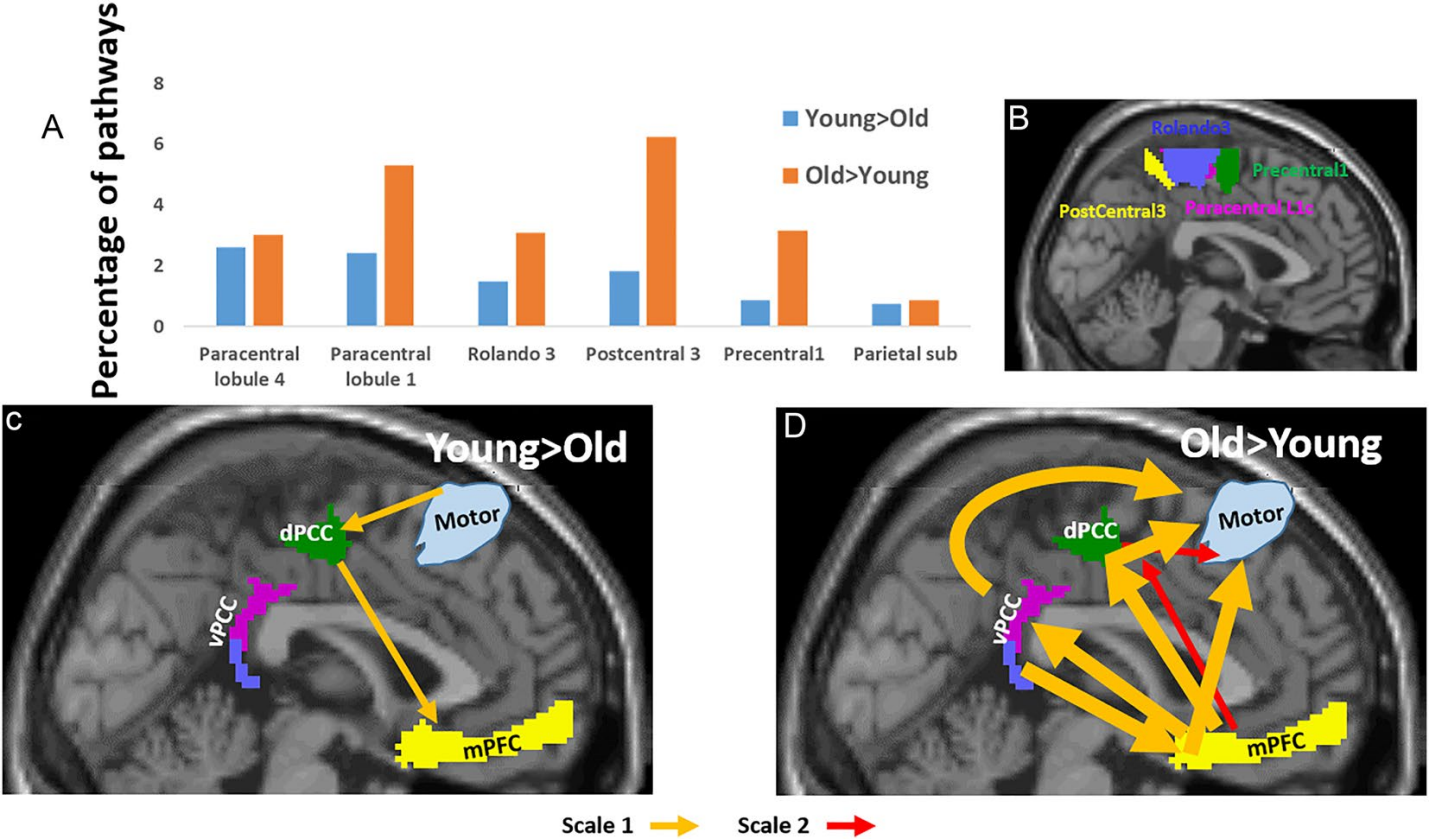
Three-node between groups functional inter-DMN-sensorimotor pathways. (**A**) Percentiles of pathways (scales 1 and 2 together) that included a sensorimotor region. In blue, pathways that were stronger in the young participants’ group, and in orange, pathways that were stronger in the older participants’ group. (**B**) A 2D sagittal projection of the three sensorimotor regions whose pathways differed most between the age groups. (**C–D**) Illustrations of the most dominant pathways that were stronger in the young participants (**C**) and were stronger in the older participants’ group (**D**), with pathways in light orange for BOLD frequency scale 1, and in red for scale 2. Only pathways whose occurrences were > 2% are shown with arrow width corresponding to pathway’s occurrences.

Table 1D and Fig. 4 gives the between-group occurrences of the 12 three-node directed pathways for pathways connecting the DMN with sensorimotor regions. Figure 4A shows that the inter-DMN-sensorimotor pathways that differed between the age groups involved mainly the postcentral, the ventral paracentral lobule, and the dorsal Rolando sulcus. Specifically, the following six AICHA regions were found: Paracentral lobule 4, Paracentral lobule 1, Rolando 3, Postcentral 3, Precentral 1 and Parietal Sub. Table 1D gives the between-group pathways’ occurrences for the inter-DMN-sensorimotor pathways. The number of pathways with positive $\Delta PW{I}^{k}\left(\omega \right)$ values was lower (26%) compared to the number of pathways with negative value ('Older > Young'). In both groups, most of the pathways were at low BOLD frequency scale (65% for Young > Older and 70% for the Older > Young pathways) and they had comparable involvements of vPCC and dPCC (61% and 59% of dPCC in Young > Older and Older > Young, respectively). However, the most significant difference between the groups was directionality: While 59% of the Young > Older pathways were directed from the sensorimotor regions towards the DMN, only 19% were in the opposite direction, while in the others the sensorimotor nodes were between the two DMN nodes. In contrast, 80% of the Older > Young pathways lead from the DMN towards the sensorimotor areas, with no pathways in the oppositely directed.

### Pathways that correlated with neuropsychological domains

To identify the pathways in the older subjects group that correlated with the neuropsychological domains, we calculated the unique contribution of each neuropsychological domain to the pathways, while statistically controlling for the contributions of all other domains as well as for age, gender, years of education and the frame-wise displacement (FD). Calculations were performed on the inter-DMN pathways (of stage 3, above). To enable comparison between pathways, we calculated the occurrences of three-node functional pathways. For that we used binary assignments, that is, pathways with a correlation value above the cutoff were assigned '1', pathways with a correlation value below minus the cutoff value were assigned '-1', and all other pathways were assigned the value zero.

**Figure 5:**
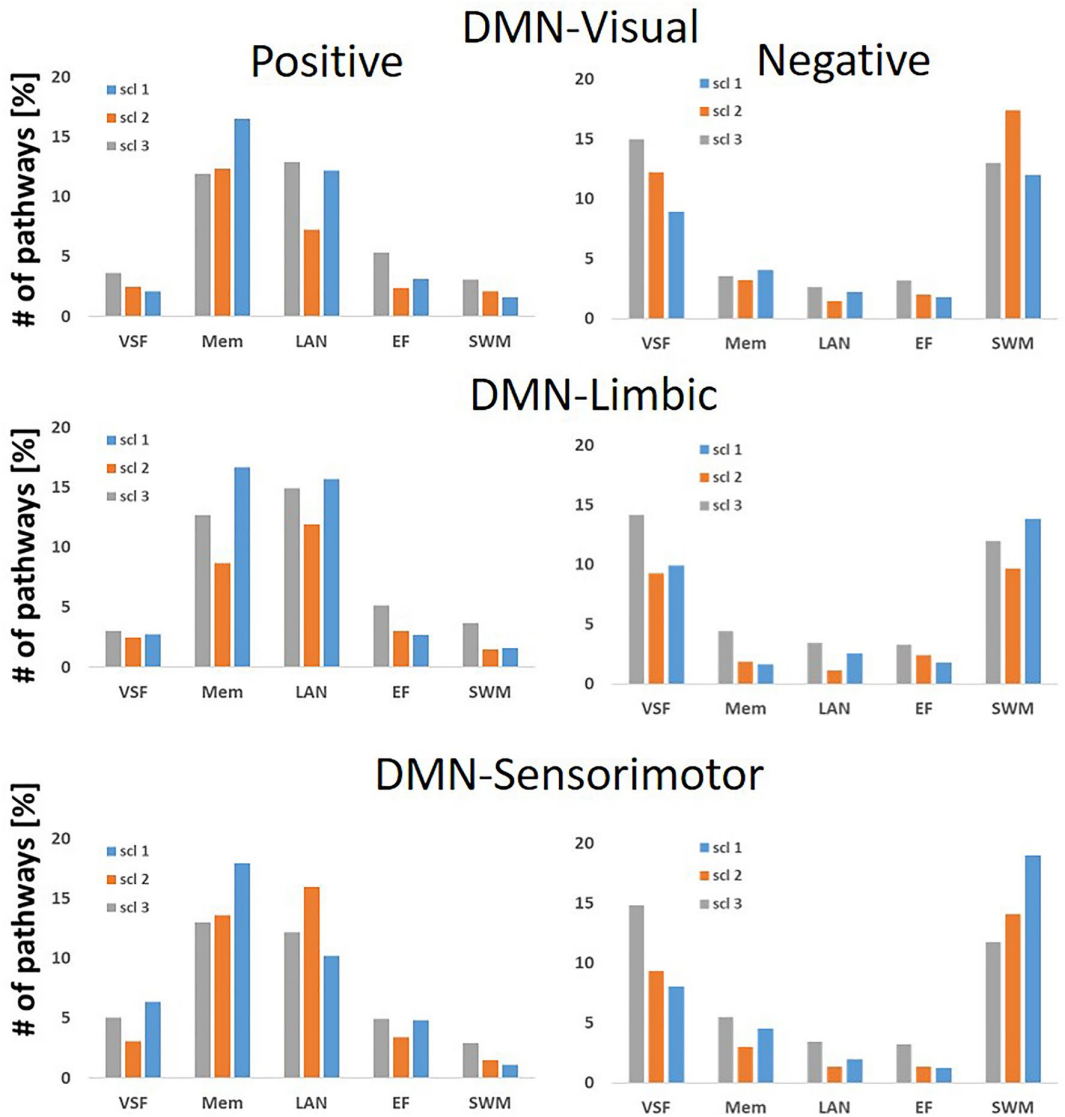
Occurrences of three-node inter-DMN pathways that correlated with the five neuropsychological domains. Top: Pathways combining the DMN with visual regions. Middle: Pathways combining the DMN with limbic regions. Bottom: Pathways combining the DMN with sensorimotor regions. Occurrences are given in percentages of the maximum possible pathway' number for Positive correlation on left and for Negative correlation on the right. VSF-Visuospatial function; Mem-Short term memory; LAN-Language; EF-Executive function; SWM-Psychomotor speed and working memory.

Supplementary Table 3 lists the occurrences of the 12 three-node directed pathways (in percentages of the maximum number) that were correlated with the five different neuropsychological domains, for inter-DMN pathways. Positive correlation implies an association between pathway’s indexes of Eq. 1 and a better score, while negative correlation relates to an association between the pathway’s indexes and a lower score. For easier observation, we present in Fig. 5 the occurrences of these pathways. The figure shows that most of the pathways with negative correlation values were with the psychomotor speed and working memory (SWM) and with the visuospatial function (VSF) domains, while most of the pathways with positive correlation values were with the language and the memory domains. Supplementary Fig. 1 presents the occurrences of the 3-node pathways that negatively correlated with SWM and VSF (all scales summed together). It shows that reduced SWM was generally correlated with pathways directed from the DMN towards non-DMN brain areas (*p* = 0.039, one side *t* test), in line with Supplementary Table 2 (*t* test between the four ‘DMN to system’ pathways of all 3 scales versus the four ‘system to DMN’ of all three scales). Supplementary Fig. 2 presents the pathways that positively correlated with language and memory for all pathways connecting the DMN with other brain regions. It shows that most of the pathways that positively correlated with memory lead from the DMN towards one of the other regions (*p* = 0.036, one side *t* test).

## Discussion

Aging is associated with dynamic changes in biological, physiological, environmental, psychological, behavioral, and social processes. The effects of aging on the brain are widespread and include among others volume shrinking, reduced dopamine and serotonin levels and vascular changes^29^. For neuroimaging macroscopic observations, we followed the line of the network degeneration hypothesis that claims that the topography of atrophy/hypometabolism follows specific brain connectivity networks^30^. We applied it to the DMN to identify degenerated or compensatory processes in older adults, using a novel method of directed connectivity previously developed by us^18–23^. By comparing directed pathways between healthy young and older subjects, we identified network alterations, and correlations of pathways with a battery of neuropsychological domains tests suggested their behavioral associations.

Our main findings were the following: (1) In older versus young participants, there were fewer pathways connecting the DMN with regions in the limbic and the visual systems, and more pathways connecting the DMN with regions in the sensorimotor system. (2) In young versus older participants, there were more pathways connecting the DMN and regions in the limbic and the visual systems that included the ventral PCC. In contrast, in older participants, these pathways included the dorsal PCC. (3) In young versus older participants, pathways between the DMN and regions within the limbic and the visual systems were at high BOLD signal frequency, while in older participants they were at low frequency. (4) Pathways connecting the sensorimotor with the DMN were sensorimotor efferent in young participants (from sensorimotor regions to the DMN), while in older participants they were sensorimotor afferent (in the opposite direction). (5) Most of the pathways that correlated with reduced psychomotor speed and working memory in older subjects were DMN efferent and (6) Most of the pathways that correlated with better memory function were also DMN efferent.

Taken together, our findings differentiated between pathways connecting regions in the sensorimotor system with the DMN and pathways connecting regions in the visual and limbic systems with the DMN. A reduced number of pathways, a shift to the dorsal PCC, and a shift to a lower frequency of the BOLD signal in older participants characterized the latter, while increased number, opposite directionality and correlation with reduced psychomotor speed and working memory characterized the former.

The shift from ventral to dorsal PCC in older participants is in line with previous reports that have shown different ventral and dorsal functional connectivity in amnestic, mildly cognitively impaired, and AD subjects^11^. Despite the extensive interconnections with memory, attention, and decision areas, the primary function of the PCC remains unknown. It has been suggested that the PCC controls the balance between internally and externally focused thoughts and might be a site involved in generating adaptive behavior to the changing world^9^. Specifically, due to its variation with learning, memory, reward, and task engagement, it was proposed that these modulations reflected the underlying processes of change detection and motivated subsequent shifts in behavior^32^. Furthermore, it was suggested that the ventral PCC supported internally directed thought^9^, while the dorsal PCC supported externally directed thought^10^. These and other findings have led to a differentiation between the dorsal and ventral parts of the PCC and to suggestions that the dorsal PCC is implicated in dynamic control of intrinsic networks and thus in attentional focus^33^. Our findings that most of the visual regions that were found in pathways that were different between the young and older subjects were within the ventral precuneus, support this account, since it has been identified as a key brain region for multiple-cue judgments. This includes such features as associative memory and inference based on analysis and rules^34^.

We infer that the BOLD signal frequency shift, in pathways combining regions in the visual and the limbic systems with the DMN, to lower frequencies in older participants, represents slower processing, which is in line with slower cognitive and limbic activities in older people^35^. This intuitive inference is based on our ongoing study using intracranial electroencephalography (iEEG) signals from the hippocampi of epileptic patients that show that processing times are inversely proportional to the signal frequency (paper in preparation). We speculate that a mapping between neural and BOLD frequencies exists, suggesting that BOLD signal frequencies are also related to processing times.

Most of the pathways that connect regions in the sensorimotor system with the DMN, were directed from the sensorimotor to the DMN in young participants and in the opposite direction in old participants. The later pathways were also correlated with reduced SWM. Detailed inspection of all pathways showed that the main differences between the groups were with the directionality of pathways involving the mPFC. Specifically, in young participants the pathways lead from the sensorimotor regions towards the mPFC and in older participants – in the opposite direction. Furthermore, in most pathways that negatively correlated with SWM (Supplementary Fig. 1C), mPFC was efferent to the sensorimotor system indicating that these processes corresponded to reduced SWM. The mPFC is critically involved in numerous cognitive functions, including attention, inhibitory control, habit formation, working memory and long-term memory. It is however not clear what were the causes for the directionality change between young and older participants and how this change was related to reduced SWM. We note that the two findings in the older group: (1) the reduced sensorimotor efferent and (2) the increased sensorimotor afferent, which seem at first sight as degenerate (the former) and compensatory (the latter) processes, were not necessarily so. The fact that the sensorimotor afferent correlated with reduced psychomotor speed and working memory made these processes difficult to consider as compensatory, since we expect compensatory processes to improve and not to disprove behavior. Therefore, we speculate that these processes were uncoupled, where the reduced sensorimotor efferent may be related to reduced physical activity, and the increased number of pathways in the reverse direction may correspond to a higher control of the mPFC on sensorimotor activity. This speculation is in line with a large body of research showing the advantages of physical activity for multiple illnesses and aging^36–38^, and with the evidence that the DMN is involved in these illnesses. The latter suggestion is based on findings of increased mPFC activity and motor control in older adults^39^.

Combining all these findings, we suggest that the shift of processes to lower BOLD signal frequencies is indicative of the reduced speed of multiple cognitive and executive processes that lead to impairments in cognitive functions^35^. The reduced sensorimotor input to the DMN, suggested as the result of reduced physical activity, and the increased need to control activity by the mPFC, causes a higher dependency on external versus internal cues, reflected by a shift from ventral to dorsal PCC of inter-DMN pathways. Consequently, we speculate that one way to slow or even reverse these processes may be by increasing physical activity, stressing the critical importance of physical activity and suggesting how it might slow aging.

## Methods

### Subjects

This study used data from young and old healthy participants who underwent fMRI measurements at two different sites using the same systems and identical protocols. The study was approved by the Ethics Committees of the Hadassah Medical Center, Jerusalem, Israel, and the General University Hospital in Prague, Czech Republic. All participants provided written informed consent prior to inclusion in the study, which was carried out in compliance with the Declaration of Helsinki.

*The young subject group*: Fifty-two undergraduate students at the Hebrew University of Jerusalem, Israel were recruited. To exclude past or present psychiatric disorders, participants were evaluated by a psychiatrist using the Structured Clinical Interview for DSM-IV (SCID-5-CV). Additional exclusion criteria were neurological disorders, and, for women, the use of hormonal contraceptives, pregnancy or breastfeeding. Two male subjects were excluded by these criteria, yielding a final sample of 20 men (age:23.9 ± 2.9 years) and 30 women (23.9 ± 2.4 years). Part of these data was used in a previous study^23,31^.

*The old subject group*: 40 elderly subjects were recruited from a community in Prague. Nine of them were excluded due to: severe atrophy or vascular lesions (n = 5), in-scanner motion (n = 3), or use of lithium (n = 1) which yielded a final sample of 31 subjects (16 females, age 61.2 ± 6.4 years, and 15 males ,65.2 ± 8.8 years). Exclusion criteria were a history of psychotic symptoms, depression, dementia or a cognitive state on the Montreal Cognitive Assessment < 22. Psychomotor speed and working memory, executive function, language, long-term memory and visuospatial function were measured (see below). Part of these data was used in previous studies^21,23,40^.

### Neuropsychological tests of older subjects

Old subjects underwent neuropsychological assessment by an experienced neuropsychologist during a preliminary visit approximately two weeks before the MRI session. The assessments included two neuropsychological tests for each of the following domains: (1) Psychomotor speed and working memory: Trail Making Test, part A^41^ and Digit span backwards from the Wechsler Adult Intelligence Scale, third revision (WAIS-III)^42^; (2) Executive function: Tower of London^43^ and semantic verbal fluency^44^; (3) Language: Boston Naming Test, Czech version^45,46^ and WAIS-III Similarities (Wechsler, 1997); (4) Long term memory: Rey Auditory Verbal Learning Test, delayed recall^47^ and Brief Visuospatial Memory Test, revised, delayed recall^48,49^; (5) Visuospatial function: CLOX^50^ and Judgment of Line Orientation^51^. The score on each test was transformed into a z-score using the Rankit formula^52^. The z-scores of each domain were summarized for each subject to create summary scores, with a higher score indicating a better function.

Table 2 lists the participants’ demographic data and the neuropsychological evaluation tests.

**Table 2:** Demographic and memory characteristics of the subjects. RAVLT- Rey Auditory Verbal Learning Test, Delayed Recall, BVMT-R—Brief Visuospatial Memory Test, Revised, Delayed Recall.

Demographic and neuropsychological variables	Young	Older
	Mean ± SD (range) (n = 50)	Mean ± SD (range) (n = 31)
Age, years	23.9 ± 2.6 (19–28)	63.2 ± 7.89 (46–83)
Gender (male/female)	20/30	15/16
Education, years	14.1 ± 1.75 (12–20)	14.8 ± 3.5 (11–25)
Long-term memory		
RAVLT-30		8.97 ± 2.5 (2–12)
BVMT-R-30		10.23 ± 1.70 (5–12)
Long-term term memory summary		67.8 ± 22.2 (8.0–94.2)
Visuospatial function		
Royall’s CLOX (CLOX I)		13.26 ± 1.15 (11–15)
Judgment of Line Orientation		24.65 ± 3.88 (11–29)
Visuospatial function summary		59.4 ± 17.9 (23.8–86.7)
Psychomotor speed and working memory		
Trail Making Test, part A		35.2 ± 9.1 (20–58)
Digit Span Backwards		6.7 ± 2.2 (4–12)
Attention summary		65.12 ± 17.4 (35.9–92.3)
Executive function		26.1 ± 4.1 (16–34)
Tower of London		65.6 ± 11.8 (38–93)
Semantic fluency		59.8 ± 18.4 (14.4–93.1)
Executive function summary		
Language		23.7 ± 5.7 (11–32)
Wechsler Adult Intelligence Scale-III Similarities		54.4 ± 6.5 (27–60)
Boston Naming Test (Czech version)		62.0 ± 13.1 (34.6–81.5)
Language summary		57.4 ± 25.6 (7–97.4)

### MRI data acquisition and preprocessing

MRI data of the young and old participants were acquired with 3T MR scanners (Magnetom Skyra, Siemens, Germany), at the neuroimaging center of the Hebrew University of Jerusalem, Israel for the young group; and at Charles University in Prague, Czech Republic for the older subjects group. At both locations, participants underwent a 10-min resting-state fMRI (rs-fMRI) during fixation on a visual crosshair, using the same MRI acquisition protocols. Functional images were acquired using a T2*-weighted gradient-echo, echo-planar imaging sequence with TR = 2 s, TE = 30 ms, image matrix = 64 × 64, field of view = 192 × 192 mm, flip angle = 90°, resolution = 3 × 3 × 3 mm, interslice gap = 0.45 mm. Each brain volume comprised 30 axial slices, and each functional run contained 300 image volumes. Anatomical images were acquired using a sagittal T1-weighted MP-RAGE sequence with TR = 2.2 s, TE = 2.43 ms, resolution = 1 × 1 × 1 mm. For the older participants group, T2-weighted images were collected as well, for diagnostic purposes.

All functional MRI data underwent the following preprocessing using SPM12 (http://www.fil.ion.ucl.ac.uk/spm/software/spm12). Functional images were spatially realigned, coregistered to T1 anatomical images; slice-time corrected and normalized to MNI space. Further preprocessing was done in a CONN toolbox^53^. Potential confounding effects were regressed out using the aCompCor method for anatomical component-based noise correction^54^. These included: (1) outlier scans, i.e. censoring/scrubbing^55^. Outlier scans were identified based on the amount of subject motion in the scanner as measured by frame-wise displacement (FD) and global BOLD signals. Acquisitions with FD > 0.45 mm or global BOLD signal changes > 5 standard deviations were considered outliers and removed by regression; (2) the first 5 principal components (PCAs) of the CSF and white matter signals, to minimize the effects of physiological non-neuronal signals such as cardiac and respiratory signals; (3) estimated subject-motion parameters and their first-order derivatives (a total of 12 parameters); (4) session effects: the potential effects of the beginning of the session were removed by a step function convolved with the hemodynamic response function, in addition to the linear BOLD signal trend. After regression of all potential confounding effects, temporal band-pass filtering (0.008–0.09 Hz) was performed. The aCompCor method^54^ was applied to regress out the first 5 principal components (PCAs) of the CSF and white matter signals. This was done to minimize the effects of potential physiological non-neuronal signals such as cardiac and respiratory signals.

### Pathway calculations

Multivariate wavelet calculations were performed with IDL version 8.2.0 (Exelis Visual Information Solutions, Inc.) using custom-developed software. The complex Morlet wavelet functions were chosen for wavelet analysis because they have been shown to provide a good trade-off between time and frequency localization^56^ and were shown to best suitable for functional MRI BOLD data^57–60^. We used 10 for the smallest scale, 2 for time resolution, and 21 scales to cover the entire frequency window. Wavelet software was provided by Torrence and Compo (http://paos.colorado.edu/research/wavelets)^26,27^. For simplicity and easier evaluation, the 21 frequencies were further averaged into three frequency bands: low (average frequency 0.022 Hz, named scale 1), intermediate (average frequency 0.04 Hz, named scale 2), and high (average frequency 0.073 Hz, scale 3).

To calculate the unique contribution of each neuropsychological domain to the pathways, we used a partial correlation model between $P{W}^{k}\left(\omega ,sub\right)$ of Eq. 1 and each neuropsychological domain, while statistically controlling for the contributions of all other neuropsychological domains as well as for age, gender, years of education and the frame-wise displacement (FD). Formally, we calculated the correlation between the residuals of $P{W}^{k}\left(\omega ,sub\right)$ of each neuropsychological domain. These residuals were obtained using linear regression with all other neuropsychological domains and with gender, age, education and FD.

### Experimental workflow

We minimized the number of pathways as follows: (1) The Atlas of Intrinsic Connectivity of Homotopic Areas (AICHA)^28^ with its 384 regions was used for region selection. The average BOLD signals from these regions for each subject were used in the analysis. (2) Pathways were calculated with three preselected regions, while the fourth was any of the other 384 regions in the atlas. That is, instead of pathways with all the possible region's combinations ($${384C}_{4}=8.9E+8),$$ we had 384 pathways for each of the three preselected regions. Note that this procedure enabled us to focus on a specific network by choosing the three preselected regions in that network.

#### Between-group pathways

Comparison between young and older participants was performed in four stages (see Supplementary Fig. 3). The first aimed at identifying pathways that were different between the groups. For that, we calculated four-node pathways with three predefined DMN regions. The three predefined regions were: one out of the two AICHA medial prefrontal cortex (mPFC) regions; one out of the three AICHA angular gyrus (Ang) regions; and one of the three AICHA posterior cingulate cortex (PCC) regions. The fourth region was any of the other AICHA regions (Supplementary Fig. 3A). Specifically, we used the AICHA ‘G_Frontal_Med_Orb-1’ and ‘G_Frontal_Med_Orb_2’ as the two mPFC regions, the ‘G_Angullar_1’, ‘G_Angular_2’ and the ‘G_Angular_3’ as the three angular regions, and ‘G_Cingulum_Post_1’, ‘G_Cingulum_Post_2’ and ‘G_Cingulum_Post_3’ as the three PCC regions. It's pertinent to note that the selection of these regions as representatives of the DMN was the only preselection step. All 18 possible combinations of the two mPFC, three Angular, and three PCC regions were utilized, allowing us to assess occurrences within these combinations. Note that the ‘G_Cingulum_Post_1’ and the ‘G_Cingulum_Pos_2’ corresponded to the ventral PCC (vPCC) while the ‘G_Cingulum_Pos_3’ corresponded to the dorsal PCC (dPCC). All preselected regions were in the dominant (left) hemisphere. Pathways were calculated for each group using Eq. 2, and their differences were calculated by Eq. 3.

In the second stage, we aimed to identify extra-DMN regions involved in many pathways that were different between the two subject groups and to categorize them (Supplementary Fig. 3B). Based on the highest occurrence at the end of the first stage, we selected 18 extra-DMN regions: six were in the visual system, six in the limbic system and six in the sensorimotor system. Importantly, it's crucial to emphasize that while our selection influenced the results, it was entirely data-driven and not influenced by any pre-existing assumptions or prior knowledge.

In the third stage, we calculated the between-groups inter-DMN four-node pathways (Supplementary Fig. 3C) defined with two regions from the DMN, one from the selected regions of the visual, limbic or sensorimotor systems and one from all other brain regions. The DMN regions were a mPFC and a PCC regions.

The fourth stage gives the final results. In this stage, we aimed to differentiate between pathways. Since four-node pathways are binary, we calculated the occurrences of three-node, between groups, functional pathways (Supplementary Fig. 3D). In these calculations, pathways with one of the two mPFC regions and pathways with one of the two vPCC were summed together, as well as pathways within the six visual/limbic or sensorimotor regions (see session 5.7).

### Pathway's occurrence

We differentiate between pathways by deriving three-nodes from the four-node pathways, and by using occurrence. Specifically, we summed the number of: (1) pathways with different fourth region, and (2) pathways with different fourth region’s location. For example, the number of a three-node pathway 'A → B → C', was obtained by the sum (occurrence) of the following four-node pathways: 'A → B → C → X'; 'A → X → B → C'; 'X → A → B → C'; and 'A → B → X → C' with X symbolized the forth regions that could be any of the AICHA regions. Consequently, the maximum number of a three-node pathway was:

$$Ma{x}_{\#}=(384-{N}_{R})\cdot \frac{4}{24}\cdot {N}_{c}$$

with 384 the number of AICHA regions, ${N}_{R}$ the number of preselected regions, 4 the number of summed pathways, 24 the number of possible pathways and ${N}_{c}$ the number of summed combinations. ${N}_{R}$ equalled 8 for the intra-DMN, and equalled 11 for the inter-DMN pathways. ${N}_{c}$ equalled 6 for the intra-DMN pathways (2 mPFC * 3 Ang) and 12 for the inter-DMN (2 mPFC * 6 regions). Note that averaging over the two vPCC regions was done in the last stage. Consequently, $Ma{x}_{\#-intra}=$ 376 and $Ma{x}_{\#-inter}=$ 746. The occurrence was presented in terms of percentiles of these maximum numbers.

### Statistical analysis

As detailed in “Experimental workflow” section and Supplementary Fig. 3, our analysis comprised four stages. Steps 1 to 3 focused on region selection, while stage 4, which involved occurrence calculations, contributed to the final conclusions. Accordingly, correction for multiple comparisons was specifically applied at this fourth stage. Our statistical approach involved setting cutoff values for the 4-node pathways in steps 1–3 for region selection and defining occurrence thresholds for the 3-node pathways in step 4.

For the 4-node pathways, we computed the null distributions of Eqs. 2 and 3 across each frequency scale. These distributions were derived from rs-fMRI signals sourced from the mPFC, angular gyrus, PCC, and precuneus of the left hemisphere, utilizing the automatic anatomical labeling (AAL) atlas^61^. To ensure independence, we employed a random number generator to select seeds from various participants. This process was iterated 10,000 times, computing Eqs. 2 and 3 each time. As anticipated, the distributions of the 24 pathways for null coupling were nearly identical. These distributions indicated that when $PW{I}^{k}\left({\omega }_{1},{\omega }_{2},{\omega }_{3}\right)$ and $\Delta PW{I}^{k}\left({\omega }_{1},{\omega }_{2},{\omega }_{3}\right)$ of Eqs. 2 and 3 equated to [0.125, 0.125, 0.33] for the young and young-old groups or [0.17, 0.17, 0.37] for the old and old-young groups, the uncorrected p-values were approximately $1{0}^{-3}$.

Regarding the occurrence cutoffs for the 3-node pathways, we utilized the 10,000 4-node null pathways mentioned above and tabulated the occurrence count of the 3-node pathways, treating these counts as null occurrences. For the 'young > older' contrast, the average null 3-node pathway count was 7.5, while 'older > young' exhibited an average of one 3-node pathway.

The uncorrected p-value for the occurrence of these 3-node pathways was computed by multiplying the p-value of the 4-node pathways by the occurrence p-value. For 'young > older,' this occurrence value was 7.5 divided by the maximum possible occurrences (376 for intra-DMN pathways and 746 for inter-DMN pathways, see “Pathway's occurrence” section). In both instances, the uncorrected p-value approximated ${10}^{-5}$, with the corrected p-value for multiple comparisons being smaller than ${10}^{-3}$.

For the partial correlation cutoff, we used similar strategy. The initial cutoff value was $\left|R\right|>0.55$ corresponded to $p=1{0}^{-3}$ and the second was 0.5% of the maximum pathway’ number.

### Pathway’s directionality

To obtain directed pathways, we had to assume what the direction of coupling was, that is, whether the signal was transferred from right to left or from left to right, in the ranked pathways of Supplementary Table 1. In other words, we had to determine whether a positive phase difference between regions *i* and *j* corresponded to the signal flow from *i* to *j* or from *j* to *i*. This factor, depends on a reference phase which is an intrinsic to the system. Since our pathways were defined for a group, the reference phase must be equal for all group’s data-sets which requires that all group’s data-sets acquired by the same system. Once this factor was assumed, we applied it to all pathways within a group^25^. To infer directionality, we needed to identify a pathway whose directionality was known or could be assumed. Similar to our previous studies^18–22,31^, we calculated thalamocortical pathways (using Eq. 2), assuming that most pathways in the resting state were bottom-up. For these calculations, the left thalamus, left primary motor and left primary sensory cortices were the preselected regions, focusing on the motor system of the dominant hemisphere, and the fourth region were rs-fMRI voxels using all 3D image voxels. Null distribution of these cases implied that the $PW{I}^{k}\left({\omega }_{1},{\omega }_{2},{\omega }_{3}\right)$ of [0.2, 0.2, 0.39] corresponded to an uncorrected p value of $p\sim 2*1{0}^{-5}$ and to corrected *p* ~ 0.001 with a cluster-size threshold of 100^22^. We found that the majority of the (undirected) pathways were either “Thalamus-M1-S1-X” or “Thalamus-S1-M1-X” with “X” presenting the clusters in the motor system or frontal areas^21,22^. Bottom-up processes suggest left-to-right directionality in the pathways of Supplementary Table 1 in the older subjects group and right to left directionality in the young group. These directionalities were applied to all the pathways in this study.

### Supplementary Information

Supplementary Information.

## Data Availability

The datasets are available from the corresponding author on reasonable request.

## Abbreviations

BOLD: Blood oxygenation level-dependent
fMRI: Functional MRI
