# Supplementary material for: Directed functional connectivity of the default-mode-network of young and older healthy subjects
Source: Sci Rep. 2024 Feb 21;14:4304. doi: 10.1038/s41598-024-54802-6 (PMC10881992; doi:10.1038/s41598-024-54802-6)
Supplement: Supplementary file 1 — Supplementary Information. [file 41598_2024_54802_MOESM1_ESM.docx]

Supplementary Information for:

“**Directed functional connectivity of the default-mode-network of healthy young and older subjects.”**

**Table 1**

| **PW number** | **Pathway** |
| --- | --- |
| 1 | R1-R2-R3-X |
| 2 | R2-R3-X-R1 |
| 3 | R3-X-R1-R2 |
| 4 | X-R1-R2-R3 |
| 5 | R1-R2-X-R3 |
| 6 | R2-X-R3-R1 |
| 7 | R3-R1-R2-X |
| 8 | X-R3-R1-R2 |
| 9 | R1-R3-X-R2 |
| 10 | R3-X-R2-R1 |
| 11 | X-R2-R1-R3 |
| 12 | R2-R1-R3-X |
| 13 | R1-X-R3-R2 |
| 14 | R2-R1-X-R3 |
| 15 | R3-R2-R1-X |
| 16 | X-R3-R2-R1 |
| 17 | R1-R3-R2-X |
| 18 | R2-X-R1-R3 |
| 19 | R3-R2-X-R1 |
| 20 | X-R1-R3-R2 |
| 21 | R1-X-R2-R3 |
| 22 | R2-R3-R1-X |
| 23 | R3-R1-X-R2 |
| 24 | X-R2-R3-R1 |

**Table 1:**. A list of all four-node pathways. R1 to R3 are three predefined regions, while X is a one of the 384 AICHA (Atlas of Intrinsic Connectivity of Homotopic Areas) regions

Inter-DMN pathways within each age group

Supplementary Table 2 depicts the one-group (young and old) occurrences of the 12 three-node directed pathways for frequency scales one and two for the pathways connecting the DMN with extra-DMN areas (refer to as 'inter-DMN' pathways, in percentage of the maximum number). Most pathways that connected the DMN with regions in the visual and the limbic systems of the young group included the ventral PCC (65% of the pathways connecting the DMN and the visual system and 45% of the pathways connecting the DMN and the limbic system). However, in the old subjects group, only 12% of the pathways connecting the DMN with the visual system, and 17% of the pathways connecting the DMN with the limbic system included the ventral PCC. No significant differences between the two age groups for pathways that included the dorsal PCC were observed. Furthermore, most of these pathways for the young group were at scale 2 (64% of the pathways connecting the DMN and visual areas and 68% of the pathways connecting the DMN and limbic areas). In contrast, most of the pathways of the old subject group were at scale 1 (69% of the pathways connecting the DMN with visual areas and 72% of the pathways connecting the DMN with limbic areas). The main difference between the groups for pathways connecting the DMN with sensorimotor regions was their directionality: 49% of the pathways were efferent to the mPFC in the old subject group, and 27% of the pathways were afferent to the mPFC in the young subject group.

| **Young** | Visual | | Limbic | | Motor | |
| --- | --- | --- | --- | --- | --- | --- |
|  | Scale 1 | Scale 2 | Scale 1 | Scale 2 | Scale 1 | Scale 2 |
| mPFC>dPCC>system |  |  |  |  |  |  |
| mPFC>vPCC>system | 9.65 | 13.94 |  | 2.55 |  | 1.74 |
| mPFC>system>dPCC |  | 1.88 |  |  |  | 1.47 |
| mPFC>system>vPCC | 3.35 | 14.61 |  | 2.82 |  | 1.88 |
| dPCC>mPFC>system | 2.41 |  | 1.07 | 1.07 |  |  |
| vPCC>mPFC>system |  |  |  |  |  |  |
| dPCC>system>mPFC | 1.21 |  |  |  | 2.95 | 1.21 |
| vPCC>system>mPFC | 1.34 | 1.47 |  |  |  |  |
| system>mPFC>dPCC |  |  |  |  |  |  |
| system>mPFC>vPCC |  | 4.83 |  | 4.42 | 1.34 | 2.41 |
| system>dPCC>mPFC |  |  | 1.07 |  | 4.83 | 1.34 |
| system>vPCC>mPFC | 2.28 |  |  |  | 2.41 |  |

| **Old** | Visual | | Limbic | | Motor | |
| --- | --- | --- | --- | --- | --- | --- |
|  | Scale 1 | Scale 2 | Scale 1 | Scale 2 | Scale 1 | Scale 2 |
| mPFC>dPCC>system |  |  |  |  | 10.46 | 5.23 |
| mPFC>vPCC>system | 4.16 | 1.47 | 1.74 |  | 3.89 | 1.74 |
| mPFC>system>dPCC | 2.28 | 1.07 | 1.47 |  | 1.34 | 2.01 |
| mPFC>system>vPCC |  | 1.21 |  |  |  |  |
| dPCC>mPFC>system |  |  |  |  |  |  |
| vPCC>mPFC>system | 1.61 |  | 1.07 |  | 8.04 |  |
| dPCC>system>mPFC |  |  |  |  |  | 2.01 |
| vPCC>system>mPFC |  |  |  |  |  |  |
| system>mPFC>dPCC | 2.01 |  |  |  |  |  |
| system>mPFC>vPCC | 1.07 |  | 1.61 |  | 1.07 |  |
| system>dPCC>mPFC |  |  |  |  |  |  |
| system>vPCC>mPFC |  |  |  |  |  |  |

**Table 2:** Percentages of a 3-node one-group DMN↔system (visual, limbic and sensorimotor). A. The young participant group. B. The old participant group. Cells with pathways of 1% of the maximum number of possible pathways or more are shown.

1. Inter DMN-Limbic Correlations

| **DMN-Limbic**  **Positive Correlations** | SWM | | | EF | | | Lan | | | Mem | | | VSF | | |
| --- | --- | --- | --- | --- | --- | --- | --- | --- | --- | --- | --- | --- | --- | --- | --- |
| Scale | 1 | 2 | 3 | 1 | 2 | 3 | 1 | 2 | 3 | 1 | 2 | 3 | 1 | 2 | 3 |
| mPFC>dPCC>system |  |  |  |  |  |  | 1.2 | 2.0 | 1.1 |  |  |  | 1.3 |  |  |
| mPFC>vPCC>system |  |  |  |  |  |  | 1.5 | 1.2 |  |  | 1.3 | 1.5 |  |  |  |
| mPFC>system>dPCC |  |  |  |  | 1.1 |  |  |  |  | 3.9 |  | 1.1 |  |  |  |
| mPFC>system>vPCC |  |  |  |  |  |  |  |  | 1.0 |  |  |  |  |  |  |
| dPCC>mPFC>system |  |  |  |  |  |  | 1.6 | 1.3 |  |  |  |  |  |  |  |
| vPCC>mPFC>system |  |  |  |  |  |  | 1.3 | 1.4 |  | 2.1 | 1.7 | 1.3 |  |  |  |
| dPCC>system>mPFC |  |  |  |  |  |  | 1.6 | 1.3 | 1.5 |  |  |  |  |  |  |
| vPCC>system>mPFC |  |  |  |  |  |  |  | 1.2 |  | 1.2 |  |  |  |  |  |
| system>mPFC>dPCC |  |  |  |  |  |  | 1.2 |  |  | 3.9 |  |  |  |  |  |
| system>mPFC>vPCC |  |  |  |  |  |  | 4.3 |  | 1.0 |  |  |  |  |  |  |
| system>dPCC>mPFC |  |  | 1.7 |  |  |  |  |  | 3.2 |  |  | 3.4 |  |  |  |
| system>vPCC>mPFC |  |  |  |  |  | 1.4 | 0.9 | 1.4 | 2.9 |  |  | 1.1 |  |  |  |

| **DMN-Limbic**  **Negative Correlations** | SWM | | | EF | | | Lan | | | Mem | | | VSF | | |
| --- | --- | --- | --- | --- | --- | --- | --- | --- | --- | --- | --- | --- | --- | --- | --- |
| Scale | 1 | 2 | 3 | 1 | 2 | 3 | 1 | 2 | 3 | 1 | 2 | 3 | 1 | 2 | 3 |
| mPFC>dPCC>system | 4.0 |  |  |  |  |  |  |  |  |  |  |  |  |  |  |
| mPFC>vPCC>system | 1.7 |  | 1.8 |  |  |  |  |  |  |  |  |  |  |  |  |
| mPFC>system>dPCC |  |  |  |  |  |  |  |  |  |  |  |  | 2.1 | 2.0 |  |
| mPFC>system>vPCC |  |  |  |  |  |  |  |  |  |  |  |  |  |  |  |
| dPCC>mPFC>system |  |  |  |  |  |  |  |  |  |  |  |  |  | 1.3 |  |
| vPCC>mPFC>system | 1.4 | 1.0 | 1.7 |  |  |  |  |  |  |  |  |  |  | 1.3 |  |
| dPCC>system>mPFC |  | 2.4 |  |  |  |  |  |  |  |  |  |  |  |  |  |
| vPCC>system>mPFC |  |  |  |  |  |  |  |  |  |  |  |  | 1.5 |  | 1.2 |
| system>mPFC>dPCC | 2.0 |  | 1.3 |  |  |  |  |  |  |  |  |  | 3.1 |  | 1.5 |
| system>mPFC>vPCC | 2.6 | 1.2 |  |  |  |  |  |  |  |  |  |  |  |  | 1.1 |
| system>dPCC>mPFC |  |  | 2.0 |  |  |  |  |  | 1.1 |  |  | 1.1 |  |  | 3.1 |
| system>vPCC>mPFC |  | 1.1 | 1.3 |  |  | 1.5 |  |  |  |  |  | 1.7 |  | 1.3 | 3.4 |

1. Inter DMN-Visual Correlations

| **DMN-visual**  **Positive Correlations** | SWM | | | EF | | | Lan | | | Mem | | | VSF | | |
| --- | --- | --- | --- | --- | --- | --- | --- | --- | --- | --- | --- | --- | --- | --- | --- |
| Scale | 1 | 2 | 3 | 1 | 2 | 3 | 1 | 2 | 3 | 1 | 2 | 3 | 1 | 2 | 3 |
| mPFC>dPCC>system |  |  |  |  |  |  |  | 1.2 |  |  |  |  |  |  |  |
| mPFC>vPCC>system |  |  |  |  |  |  | 1.7 |  |  | 2.3 | 1.6 | 1.9 |  |  |  |
| mPFC>system>dPCC |  |  |  |  |  |  |  |  |  | 2.8 | 2.7 | 1.6 |  |  |  |
| mPFC>system>vPCC |  |  |  |  |  |  | 1.3 |  |  |  |  | 1.4 |  |  |  |
| dPCC>mPFC>system |  |  |  |  |  |  | 1.2 |  |  |  |  |  |  |  |  |
| vPCC>mPFC>system |  |  |  |  |  |  |  | 1.0 |  | 2.9 | 2.0 | 1.1 |  |  |  |
| dPCC>system>mPFC |  |  |  |  |  |  | 1.1 |  |  | 2.4 |  |  |  |  |  |
| vPCC>system>mPFC |  |  |  |  |  |  |  | 0.7 | 1.7 | 1.8 |  |  |  |  |  |
| system>mPFC>dPCC |  |  |  |  |  |  |  |  |  | 1.2 | 2.3 |  |  |  |  |
| system>mPFC>vPCC |  |  |  |  |  |  | 1.7 |  |  |  | 1.0 | 1.0 |  |  |  |
| system>dPCC>mPFC |  |  |  |  |  |  | 1.5 |  | 2.5 |  |  | 1.1 |  |  |  |
| system>vPCC>mPFC |  |  |  |  |  | 1.4 |  | 1.2 | 3.5 |  |  | 1.3 |  |  | 1.1 |

| **DMN-Visual**  **Negative Correlations** | SWM | | | EF | | | Lan | | | Mem | | | VSF | | |
| --- | --- | --- | --- | --- | --- | --- | --- | --- | --- | --- | --- | --- | --- | --- | --- |
| Scale | 1 | 2 | 3 | 1 | 2 | 3 | 1 | 2 | 3 | 1 | 2 | 3 | 1 | 2 | 3 |
| mPFC>dPCC>system | 1.5 | 4.0 | 1.2 |  |  |  |  |  |  |  |  |  | 1.2 |  |  |
| mPFC>vPCC>system | 1.4 | 2.5 | 2.5 |  |  |  |  |  |  |  |  |  | 1.1 |  |  |
| mPFC>system>dPCC | 3.4 |  |  |  |  |  |  |  |  |  |  |  | 1.3 | 1.3 | 1.6 |
| mPFC>system>vPCC |  |  |  |  |  |  |  |  |  |  |  |  |  |  |  |
| dPCC>mPFC>system |  |  |  |  |  |  |  |  |  |  |  |  |  |  |  |
| vPCC>mPFC>system | 1.6 |  |  |  |  |  |  |  |  |  |  |  |  | 1.0 |  |
| dPCC>system>mPFC |  | 4.0 |  |  |  |  |  |  |  |  |  |  | 1.2 |  |  |
| vPCC>system>mPFC |  |  |  |  |  |  |  |  |  |  |  |  | 1.5 |  |  |
| system>mPFC>dPCC | 2.3 |  | 1.2 |  |  |  |  |  |  |  |  |  |  |  | 2.5 |
| system>mPFC>vPCC |  | 1.8 |  |  |  |  |  |  |  |  |  |  |  | 2.5 |  |
| system>dPCC>mPFC |  |  | 2.1 |  |  |  |  |  |  |  |  |  |  | 2.1 | 3.4 |
| system>vPCC>mPFC |  | 1.3 | 1.8 |  |  |  |  |  |  |  |  | 1.9 |  | 1.5 | 3.4 |

1. Inter DMN-Sensorimotor Correlations

| **DMN-SM**  **Positive Correlations** | SWM | | | EF | | | Lan | | | Mem | | | VSF | | |
| --- | --- | --- | --- | --- | --- | --- | --- | --- | --- | --- | --- | --- | --- | --- | --- |
| Scale | 1 | 2 | 3 | 1 | 2 | 3 | 1 | 2 | 3 | 1 | 2 | 3 | 1 | 2 | 3 |
| mPFC>dPCC>system |  |  |  | 2.0 | 1.7 |  |  | 1.1 |  | 7.0 | 2.1 | 1.1 | 4 |  |  |
| mPFC>vPCC>system |  |  |  |  |  |  | 1.6 |  | 1.1 | 2.0 | 5.8 | 2.4 |  |  |  |
| mPFC>system>dPCC |  |  |  |  |  |  |  | 2.3 | 1.7 |  | 2.3 | 1.5 |  |  |  |
| mPFC>system>vPCC |  |  |  |  |  |  |  |  |  |  |  |  |  |  |  |
| dPCC>mPFC>system |  |  |  |  |  |  |  |  |  |  |  |  |  |  |  |
| vPCC>mPFC>system |  |  |  |  |  |  | 1.3 |  |  | 4.3 |  |  |  |  |  |
| dPCC>system>mPFC |  |  |  |  |  |  | 1.7 | 5.0 |  |  |  |  |  |  |  |
| vPCC>system>mPFC |  |  |  |  |  |  |  | 1.0 |  | 1.2 |  |  |  |  |  |
| system>mPFC>dPCC |  |  |  |  |  |  |  |  |  |  |  |  |  |  |  |
| system>mPFC>vPCC |  |  |  |  |  |  |  | 1.0 |  |  |  |  |  |  |  |
| system>dPCC>mPFC |  |  |  |  |  | 1.5 | 2.1 |  | 2.9 |  |  | 1.9 |  |  |  |
| system>vPCC>mPFC |  |  |  |  |  |  |  | 2.7 | 1.9 |  |  | 2.1 |  |  |  |

| **DMN-SM**  **Negative Correlations** | SWM | | | EF | | | Lan | | | Mem | | | VSF | | |
| --- | --- | --- | --- | --- | --- | --- | --- | --- | --- | --- | --- | --- | --- | --- | --- |
| Scale | 1 | 2 | 3 | 1 | 2 | 3 | 1 | 2 | 3 | 1 | 2 | 3 | 1 | 2 | 3 |
| mPFC>dPCC>system | 8.7 | 2.7 | 1.1 |  |  |  |  |  |  |  |  |  | 2.3 | 1.3 | 1.6 |
| mPFC>vPCC>system | 3.8 | 1.7 | 1.9 |  |  |  |  |  |  |  |  |  |  |  | 1.1 |
| mPFC>system>dPCC | 1.7 |  |  |  |  |  |  |  |  |  |  |  | 1.2 | 2.3 |  |
| mPFC>system>vPCC |  |  |  |  |  |  |  |  |  |  |  |  |  |  |  |
| dPCC>mPFC>system |  |  |  |  |  |  |  |  |  |  |  |  |  |  |  |
| vPCC>mPFC>system | 2.3 | 1.2 |  |  |  |  |  |  |  |  |  |  | 1.5 |  | 1.7 |
| dPCC>system>mPFC |  | 2.8 | 1.2 |  |  |  |  |  |  |  |  |  |  |  |  |
| vPCC>system>mPFC |  |  |  |  |  |  |  |  |  |  |  |  | 1.1 |  | 1.6 |
| system>mPFC>dPCC |  |  |  |  |  |  |  |  |  |  |  |  |  |  | 1.1 |
| system>mPFC>vPCC |  | 1.1 | 2.3 |  |  |  |  |  |  |  |  |  |  |  |  |
| system>dPCC>mPFC |  |  | 2.1 |  |  |  |  |  |  |  |  |  |  |  | 2.7 |
| system>vPCC>mPFC |  | 1.3 | 1.1 |  |  |  |  |  |  |  |  | 1.7 |  | 1.0 | 3.2 |

**Table 3:** Percentages of a 3-node DMN↔system pathways of the old subject group that correlated with neurophysiological categories. Cells with pathways of >1% of the maximum number of possible pathways, are shown.


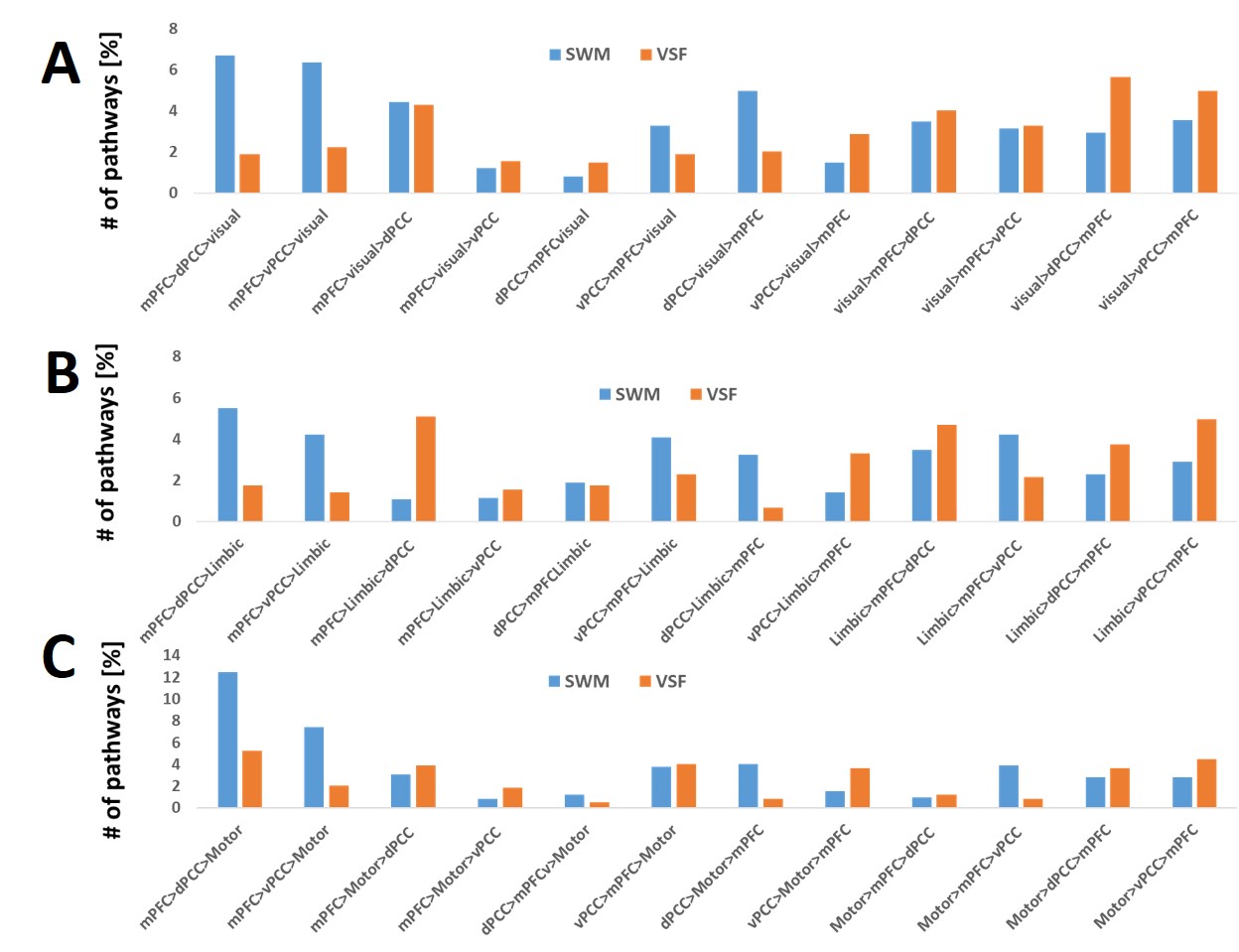


Figure 1: Occurrences of the three-node inter-DMN pathways that negatively correlated with psychomotor speed and working memory and with visuospatial function. Occurrences of the 12 possible pathway' permutations are shown. A. The inter DMN-visual pathways. B. The inter DMN-Limbic pathways. C. the inter DMN-sensorimotor pathways. Note that occurrences present the sum of all three frequency scales.


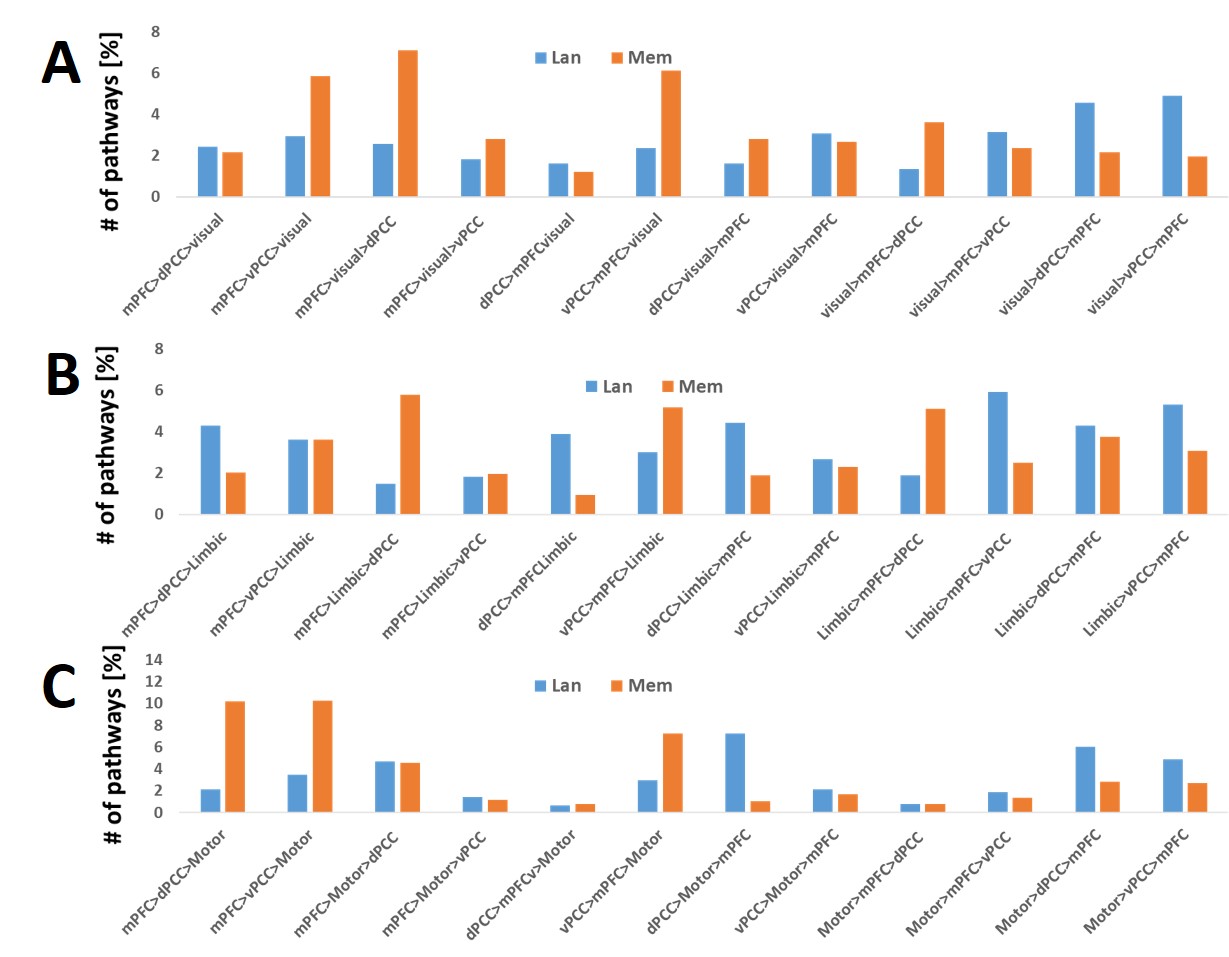


Figure 2: Occurrences of the three-node inter-DMN pathways that positively correlated with language and memory. Occurrences of the 12 possible pathway' permutations are shown. A. The inter DMN-visual pathways. B. The inter DMN-Limbic pathways. C. the inter DMN-sensorimotor pathways. Note that occurrences present the sum of all three frequency scales.


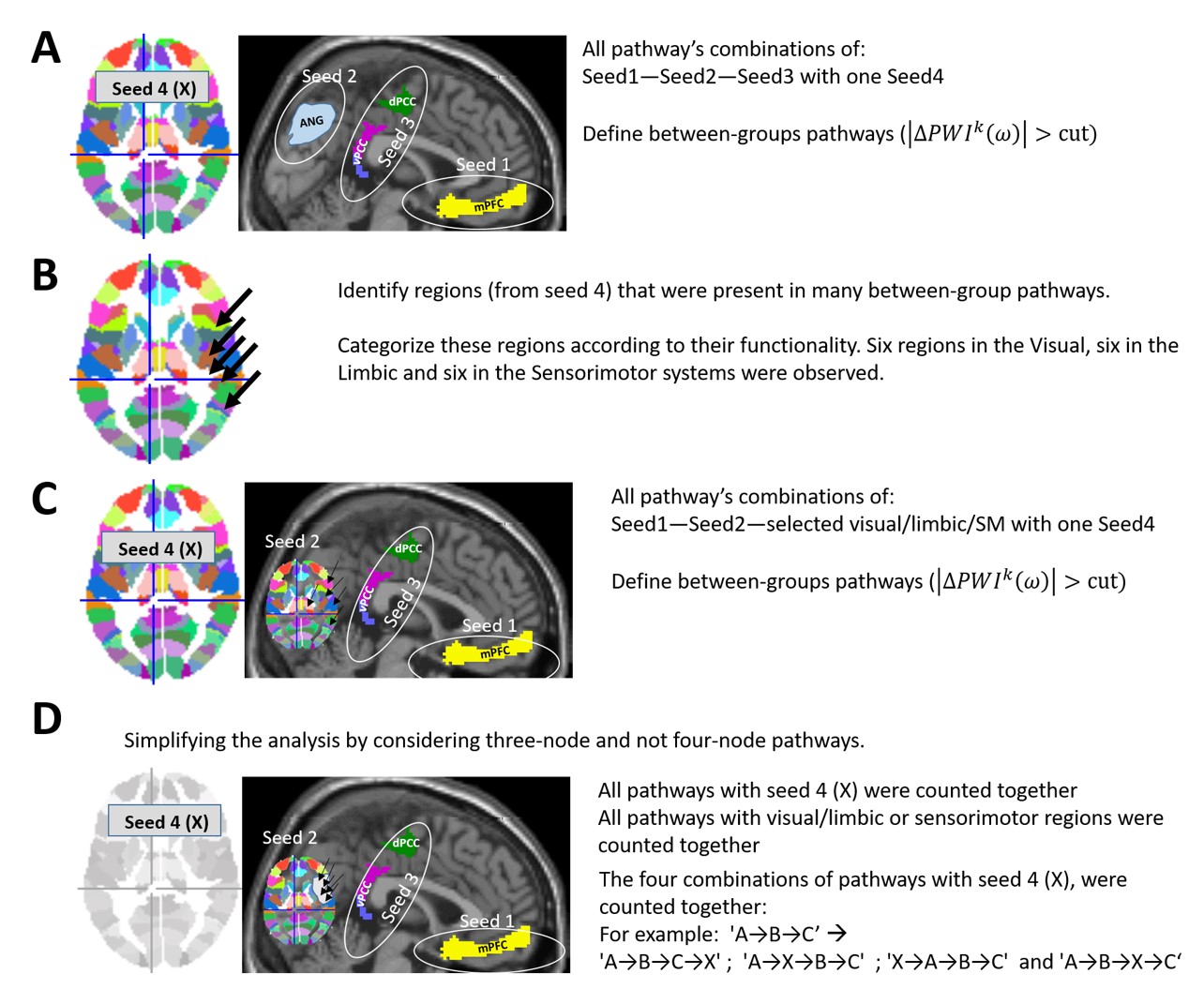


Figure 3: Illustration of the workflow. **A**. Calculations of all between-group four-node pathway combinations with three predefined DMN regions and another brain region. **B**. We identified 18 non-DMN regions that were in many between-group pathways: six from the visual, six from the limbic and six from the sensorimotor systems. **C**. Calculating all between-group four-node pathway combinations using two DMN predefined regions, one predefined region from the regions selected in B, and one free region. **D**. To enable comparison between pathways, we calculated the occurrences of three-node between-group functional pathways (calculated in A and in C) by summing together the pathways of a different fourth seed and pathways with different fourth seed locations. These three-node pathways are presented in the text.
